# The Influence of Water-Unextractable Arabinoxylan and Its Hydrolysates on the Aggregation and Structure of Gluten Proteins

**DOI:** 10.3389/fnut.2022.877135

**Published:** 2022-04-08

**Authors:** Juan Sun, Xiaojing Si, Tingting Li, Jiajia Zhao, Haifeng Qian, Yan Li, Hui Zhang, Xiguang Qi, Li Wang

**Affiliations:** ^1^State Key Laboratory of Food Science and Technology, School of Food Science and Technology, National Engineering Research Center for Functional Food, Jiangnan University, Wuxi, China; ^2^Department of Food Science and Engineering, College of Light Industry and Food Engineering, Nanjing Forestry University, Nanjing, China; ^3^College of Cooking Science and Technology, Jiangsu College of Tourism, Yangzhou, China

**Keywords:** water-unextractable arabinoxylan, gluten, chemical interactions, hydrolyzed arabinoxylan, thermal process

## Abstract

This study aimed to investigate the influence of water-unextractable arabinoxylan (WUAX) and its hydrolysates on the aggregation and structure of gluten proteins and reveal the underlying mechanism. In this work, the WUAX was treated with enzymatic hydrolysis and the changes of their molecular weights and structures were analyzed. Meanwhile, the conformation and aggregation of gluten were determined by reversed-phase HPLC, FT-Raman spectroscopy, and confocal laser scanning microscopy. The results showed that the extra WUAX could impair the formation of high Mw glutenin subunits, and the enzymatic hydrolysis arabinoxylan (EAX) could induce the aggregation of gluten subunits. And, the gluten microstructure was destroyed by WUAX and improved by EAX. Besides, the interactions of WUAX and EAX with gluten molecules were different. In summary, these results indicated that enzymatic hydrolysis changed the physicochemical properties of arabinoxylan and affected the interaction between arabinoxylan and gluten proteins.

## Introduction

Wheat gluten protein is an important functional component in flour, accounting for 85% of total wheat protein, which is composed of a variety of complex protein mixtures (1). Hydrated gluten protein determined the viscoelasticity of dough processing, and its properties affected the quality of final dough products (2, 3). Gluten proteins can be broadly classified as glutenin and gliadin (4). The elasticity of dough is influenced by glutenin, while the viscosity and ductility of dough are mainly determined by gliadin. The structural and molecular characteristics of glutenin and gliadin gave the wheat-based products a unique structure and quality (5, 6). During dough mixing, the gluten network was formed by the intermolecular and intramolecular S-S bonds, hydrogen bonds, and hydrophobic interactions between polypeptide chains by polymerization of glutenin and glutenin (7).

The processing and production of cereals produced large amounts of inedible waste such as grain bran, which was often used to make animal feed (8). These wastes often contained many different types of non-starch polysaccharides, including hemicellulose, cellulose, and other materials such as lignin and pectin (9). Arabinoxylan (AX), as important hemicellulose, accounted for about 15–22% of the wheat bran (10). It was reported that AX helped to reduce blood cholesterol, regulate blood sugar levels, improve metabolic syndrome, and regulate intestinal microorganisms, so it had attracted more and more attention. According to the solubility of AX in water, it could be classified as water-extractable arabinoxylan (WEAX) and water–unextractable arabinoxylan (WUAX) (11). In general, WUAX content in wheat bran was more than 90% of the total content of AX (12).

The molecular and structural characteristics of AX determined that its presence had a strong effect on the processing properties of the dough. Li et al. (13) found that AX gel competed with the gluten matrix for water in the whole-wheat dough system, resulting in water migration to AX gel, which led to a decline in the quality of whole-wheat bread baking. Frederix et al. (14) found that WUAX hampered the aggregation of gluten protein in the dough development process. Meanwhile, the WUAX reduced the viscoelasticity of gluten and formed a softer gluten system (15). The results showed that the gluten network of rye dough increased by 38% with the addition of enzyme preparation, while the volume of bread was increased by 10.67%. Yang et al. (16) found that the synergistic effects of xylanase and glucose oxidase significantly increased the content of free sulfhydryl groups and gluten macropolymer and improved the dough properties, which were dependent on the degree of hydrolysis of WUAX. Buksa et al. (17) found that the addition of hydrolyzed AX increased water absorption, the increasing content of AX was accompanied by the increase of bread volume and the decrease of bread core hardness.

Our previous research confirmed that WUAX had a positive effect on the stability and water retention of dough, but the texture of dough after heating was not satisfactory (15, 18). At the same time, the addition of xylanase had a very positive effect on the specific volume and texture of whole-wheat steamed bread (19). Thus, we speculated that this change was mainly due to the different effects of AX with and without enzymolysis on the gluten aggregation behavior. At present, a large number of relevant studies mainly focused on the dough development stage or the product properties, but few studies on the interaction between AX and gluten protein in the heating stage. Therefore, it is important to study the properties of AX with and without enzymatic hydrolysis on the specific mechanism of thermal polymerization of gluten that was of great significance for the subsequent application of AX in wheat-based foods (19). It was found that most WUAX harmed the quality of grain products, and the enzymatic hydrolysis of xylanase could usually improve these negative effects, but the mechanism was still unclear.

To sum up, we hypothesized that the negative influences of WUAX on functional characteristics of gluten could be alleviated by enzymolysis of xylanase. Its change might be due to the difference in the effects of AX with or without enzymolysis on the gluten aggregation behavior. We determined the effect of enzymatic hydrolysis on molecular weight and chain structure of WUAX to analyze the changing mechanism. To reveal the effects of WUAX with or without enzymolysis on the conformation and aggregation of gluten during thermal treatment, different subunits distribution, aromatic amino acid microenvironment, and characterization of gluten microstructure were determined. Meanwhile, the interactions of WUAX with or without enzymolysis between gluten molecules were evaluated by the fluorescence quenching method. This manuscript was to reveal the potential mechanism of enzymatic hydrolysis of WUAX to improve the functional properties of gluten protein during heating treatment.

## Materials and Methods

### Materials

The wheat gluten (Lot EOTZB) was purchased from TCI Chemical Co. (Nihonbashi-Honcho, Chuo-ku, Tokyo, Japan). The composition of wheat gluten was measured, using the approved methods of the AACC International (2000, methods 46-13, 30-10, 76-13, 44-19, and 08-01) and the results were expressed as average values. Protein, fat, starch, moisture, and ash contents were 72.96, 6.1, 4.9, 4.6, and 4.4%, respectively. Wheat bran was obtained from Yihai Kerry Co., Ltd. (Kunshan, China). The remaining chemicals and the solvents used were of analytical quality.

### Exaction and Hydrolyzation of Arabinoxylan

The alkali extraction process of WUAX was carried out according to our previous methods (15). The 5% WUAX solution (w/v) was prepared with 25 mM sodium acetate buffer (pH 5.0). After the solution was evenly distributed in a water bath at 55°C, 0.1% xylanase (w/w, WUAX dry weight) (6,000 IU/mg) was added and hydrolyzed for 6, 12, 18, and 24, respectively. Then, the enzyme was destroyed in a boiling water bath for 10 min, freeze-dried, ground, and screened for use. The sample after enzymatic hydrolysis was called EAX (enzymatic hydrolysis arabinoxylan), and the number after represents the time of enzymatic hydrolysis.

### Size-Exclusion Chromatography

Samples were prepared according to the method reported by Yadav et al. (20). In conclusion, the mobile phase was 0.05 M NaNO_3_ solution (containing 0.01% NaN_3_), 0.45 μm water system filtration membrane, and ultrasonic degassing for 30 min. The solutions of WUAX or EAX (5 mg/mL) were prepared in the mobile phase. Samples were dissolved overnight at 4°C and filtered through a 0.45 μm water system filter. The solvent delivery system consisted of a Waters 2695 degassing unit, a manual sampler, a pump, and a Waters 2489 UV detector. The flow rate of the mobile phase was 0.6 mL/min. The samples were injected through a 300 μL full loop, in triplicate. The chromatograph includes Dawn Heleos II multi-angle laser light scattering photometer (MALLS). Data were processed by Astra v 5.3.4 software.

### Fourier Transform Infrared Spectroscopy Determination

The infrared spectra of samples were measured by Antaris II Fourier transform near-infrared spectroscopy (8). The AX and KBr powder were uniformly mixed and ground in an agate mortar, then pressed into a transparent slice. The infrared spectroscopy of the sample was scanned with air as the background, the spectral scanning range was from 4,000 to 400 cm^–1^ with a resolution of 4 cm^–1^ for 64 scans.

### Preparation of Wheat Gluten Samples

The powder of the gluten sample (1.0 g) was premixed with different concentrations of WUAX or EAX (0, 3, 6%, w/w, per gram of gluten), followed by the addition of 5.0 mL deionized water. Excess water was added to get rid of the impact of water content. The sample was kneaded with a spatula till the formation of a uniform dough. Then, the uniform dough was completely hydrated at 4°C for 1 h. The samples were abbreviated as G for native gluten (without WUAX), GW for gluten–WUAX, and GE for gluten-EAX. The content of WUAX was written as a number after the abbreviated letters. Subsequently, samples were held for 30 min at 25, 60, and 95°C. Samples were immediately cooled in liquid nitrogen after heating. All samples were then lyophilized for further analysis.

### Chemical Composition Analysis

The monosaccharide composition of WUAX was determined according to Stoklosa et al. (21). A 5 mg sample was weighed and dissolved in 2 mL 2 M trifluoroacetic acid solution in a hydrolysis tube. The samples were sealed with Teflon screw caps with an outer O ring, hydrolyzed at 121°C for 2 h, and cooled to room temperature. The samples’ nitrogen was blown to dry, then distilled water was added to dissolve the residue, the solution was up to a constant volume of 50 mL, and was then diluted 10 times. The hydrolyzed solution passed through a 0.22 μm water system filter. The analysis was performed using an Agilent 1260 Infinity II high-performance liquid chromatograph equipped with a refractive index (RI) detector. The mobile phase was ultrapure water. The reference substances containing arabinose, xylose, glucose, galactose, glucuronic acid, and galacturonic acid were analyzed simultaneously with the sample.

### Reversed-Phase High-Performance Liquid Chromatography

According to the method of Chen et al. (22) with some modifications, the contents of gliadin and glutenin subunits in gluten samples were determined by reversed-phase high-performance liquid chromatography (RP-HPLC). The gluten (100 mg) was extracted twice with 1.0 mL of 60% (v/v) ethanol at room temperature for 15 min, then the supernatant was extracted by centrifugation (12,000 × *g*, 10 min, 20°C) to obtain the gliadin fraction. The precipitation was extracted with 1.0 mL solution containing 50% (v/v) propylene glycol, 2 M urea, 1% (w/v) dithiothreitol, and 0.05 M Tris-HCl at 60°C for 45 min. The supernatant was extracted by centrifugation (12,000 × *g*, 10 min, 20°C), and the precipitation was repeated twice. The supernatant was combined to obtain the gluten component. All extracts were filtered by a 0.22 μm organic filter. The samples were analyzed by Prominence Shimadzu high-performance liquid chromatography. The sample size was 10 μL and the column was Aeris Widepore XB-C18 column (3.6 μm, 150 × 4.6 mm, Phenomenex). The elution solvents were ultra-pure water (A) containing 0.1% trifluoroacetic acid and acetonitrile (B) containing 0.1% trifluoroacetic acid. The linear gradient was 0 min 24% B∼50 min 56% B, the flow rate was 0.6 mL/min, column temperature was 60°C, and detection wavelength was 210 nm. Gliadin and gluten were identified by comparison with standard retention times and spectra of proteins, while quantitative analysis was based on their chromatographic peak area. All samples were in triplicate and the average of the three measurements was taken.

### Fourier Transform Raman Spectroscopy

According to the methods described by Rygula et al. (23) with some modifications, the Raman spectra of gluten protein samples were scanned. The powder sample was placed on a microscope slide covered with tin foil. Raman spectra were obtained at 532 nm laser wavelength, 10 mW laser output power, and 10 mW laser energy. The scanning range was 400–3,500 cm^–1^.

### Confocal Laser Scanning Microscopy

Following the method of Wang et al. (24) with slight modifications, the gluten dough was prepared as described in the preparation of wheat gluten samples, but part of the water had a fluorescent brightener-28 dye (2.00 mL; 0.1 mg/mL) and Rhodamine B dye (0.43 mL; 0.1 mg/mL) so that the dough contained 10 PPM of fluorescent brightener −28 and Rhodamine B (based on gluten protein). After the dough was processed, it was cut into 5 mm × 5 mm × 5 mm squares, put into the mold, and wrapped with OCT embedding agent, and then transferred to the −80° refrigerator. The dough was cut into thin slices of 10 μm using a Leica CM1950 frozen slicer. The dough slices were adsorbed on the microscope slides and fixed with methanol. The sample was observed on LSM880 high-resolution confocal laser microscope with a 20× objective lens. The images were scanned sequentially with different laser beams and emission filters. The fluorescence brightener-28 was excited at 405 nm and detected at 410–470 nm. Rhodamine B was excited at 561 nm, and the detection wavelength ranged from 575 nm to 620 nm. A 512 × 512 pixel image was taken at a speed of 7 μs/pixel.

### Fluorescence Spectroscopy and Thermodynamics

The intrinsic fluorescence intensity of gluten protein would change when it was combined with AX. The fluorescence quenching analysis of the interaction between gluten protein and AX sample was carried out according to the method of Shamsi et al. (25) with some modifications. The intrinsic fluorescence spectra of 0.2 mg/mL protein solution were measured at 20, 25, 30, 55, 60, 65, 90, 95, and 100°C by adding different AX solutions (final concentration was 0–2 μmol/L). The excitation wavelength was 280 nm, and the spectral scanning range was 300∼450 nm. Different mathematical equations were used to analyze the fluorescence quenching data, and equation 1 was used to determine the quenching mechanism between the quenching agent and the gluten protein:


(1) $$\frac{F_{0}}{F}=1+K_{q}\,\tau_{q}\,\left[Q\right]=1+K_{S\,V}\,\left[Q\right]$$

where, F_0_ and F are fluorescence intensities in the presence and absence of quenching agent (AX), respectively; [Q] denotes the concentration of quenching agent (AX); K_*q*_ is the quenching rate constant of protein; τ_*q*_ is the average lifetime of tryptophan in the absence of quenching agent (10-8 s); K_*SV*_ is the quenching constant.

The apparent binding constant K_*b*_ and binding site n were determined as Equation 2.


(2) $$l\,o\,g\,\frac{F_{0}-F}{F}=l\,o\,g\,K_{b}+n\,l\,o\,g\,\left[G\right]$$

Thermodynamic parameters were determined by Equation 3.


(3) $$I\,n\,K_{b}=-\frac{△\,H}{R\,T}+\frac{△\,S}{R}$$

In the formula, R is the ideal gas constant, 8.314 J⋅K-1⋅mol-1; T is the experimental temperature; ΔH is enthalpy change; ΔS is the entropy change.

ΔG was calculated by Equation 4.


(4) △⁢G=△⁢H-T⁢△⁢S

### Statistical Analysis

The data were processed by SPSS statistics software (version 25.0, SPSS Inc., Chicago, IL, United States) and the results were presented as mean ± standard deviation (SD). Multivariate mean analysis was performed by one-way analysis of variance (ANOVA). The difference was statistically significant (*P* < 0.05).

## Results and Discussion

### Chemical Composition and Molecular Weight Analysis

The alkali extracted method which we used was to maximize the extraction of the AX from wheat bran. The results showed the yield and purity were 13.24 and 87.65%, respectively. The monosaccharide composition analysis presented that there were four different monosaccharides, including arabinose, galactose, glucose, and xylose, which content were 27.59, 1.63, 4.44, and 52.41%, respectively. It was obvious that the arabinose and xylan were the main monosaccharides of WUAX. Furthermore, the arabinose/xylose ratio (Ara/Xyl) of the WUAX was 0.53. Similarly, Aguedo et al. (26) found that the Ara/Xyl of wheat bran was 0.54, which indicated that our alkali extracted method had less damage to the structure of the side chains of WUAX. Based on our previous study, WUAX was hydrolyzed by endo-1, 4-β-xylanase for different times for further analysis. The molecular weight distribution of untreated and enzymatically hydrolyzed arabinoxylans was determined by size-exclusion chromatography. As presented in Table 1, WUAX only had one main peak with a molecular weight of 450 kDa. While, the polydispersity index (PDI, Mw/Mn) of WUAX was 4.5, indicating that the AX extracted from wheat bran had different molecular weights and chain structures (27). After the hydrolysis by endo-1, 4-β-Xylanase, all the EAXs had two different main peaks. Evidently, the molecular weight of AX decreased compared with the WUAX, while the molecular weight distribution among EAXs changed a little. Furthermore, after a long time of enzymatic hydrolysis, the molecular weight loss was limited, which was due to the limited capacity to hydrolyze the long-chain arabinoxylan.

**TABLE 1:** The molecular weight distribution of untreated and enzymatically hydrolyzed arabinoxylans.

Samples	Peak 1			Peak 2		
	Proportion (%)	Mw (kDa)	Mn (kDa)	Proportion (%)	Mw (Da)	Mn (Da)
WUAX	100.0	450	100	NA	NA	NA
EAX-6	58.3	420	120	29.3	6520	3570
EAX-12	58.9	320	70	41.1	2540	930
EAX-18	61.0	300	60	39.0	2380	1080
EAX-24	36.2	390	100	63.8	3790	770

*Mw, weight average molecular weight; Mn, number average molecular weight; WUAX, water-unextractable arabinoxylan; EAX-6, EAX-12, EAX-18, EAX-24, enzymatic hydrolysis arabinoxylan (the WUAX hydrolyzed by xylanase for 6, 12, 24, and 24 h, respectively).*

**FIGURE 1:**
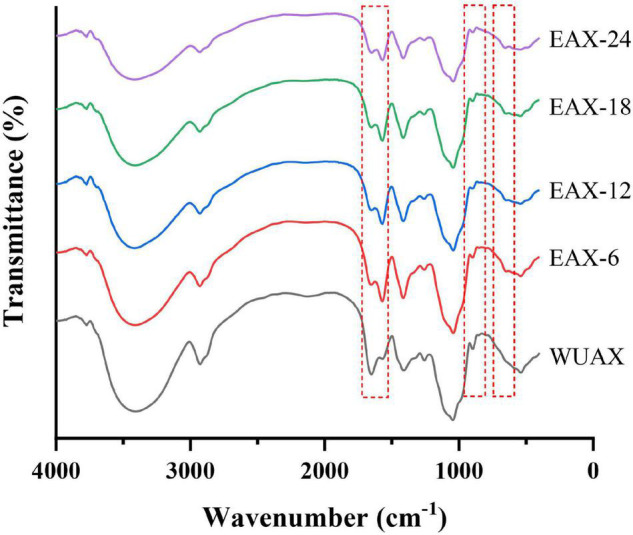
The FTIR spectrum of WUAX and EAX. WUAX, Water-unextractable arabinoxylan; EAX6, EAX12, EAX18, EAX24, enzymatic hydrolysis arabinoxylan (the WUAX hydrolyzed by xylanase for 6, 12, 24, and 24 h, respectively).

The FTIR spectrum of WUAX and its enzymatic hydrolysates were presented in Figure 1. The stretching vibration of hydroxyl groups was in the range of 3,600–3,200 cm^–1^, this was due to the hydrogen bond interactions among polysaccharides. A broad and single peak was observed at about 3,450 cm^–1^, indicating the presence of intramolecular hydrogen bonds. A small band observed at 2,910 cm^–1^ was attributed to the asymmetric stretching vibration of carbon-hydrogen bonds (28). The bands near 1,650 cm^–1^ were corresponding to asymmetric COO-, representing the presence of the free carboxylic acids and uronic acids in WUAX. The weak peak nearly about 1,510 cm^–1^ was the characteristic of the bending/stretching of lignin aromatic hydrocarbons. From Figure 1, after enzymatic hydrolysis, the band intensity near 1,650 cm^–1^ decreased and that of around 1,510 cm^–1^ increased. This phenomenon indicated that the WUAX with long chains and complex structures could aggregate and reunite with each other, thus the enwinding of polysaccharide molecular chains resulted in the mask of some groups (8). The enzymatic hydrolysis cleaved the chains of the polysaccharide molecules, then the aggregation and enwinding of molecular chains were reduced, and the internal groups of the molecules were exposed. Meanwhile, the conditions of enzymatic hydrolysis (at about pH 5) might have led to the hydrolysis of uronic acid in the polysaccharide, resulting in a decrease of band intensity. A relatively strong peak near 1,030 cm^–1^ indicated the bending of C-OH (29). And the weak shoulder at 993 cm^–1^ was characteristic of arabinoxylan-type polysaccharides. Besides, the characteristics of β-linkage of pyranose were observed near 890 cm^–1^ (30).

### Different Subunits Distribution

The typical RP-HPLC chromatogram is a useful tool for illustrating changes in gluten subunits distribution with different AXs at different heating temperatures. The determined and analyzed results of different subunits distribution were presented in Table 2. The typical gliadin subunits can be divided into ω 5-, ω 1, 2-, α-, γ-gliadin according to retention time (31). It can be seen that the number of total gliadins decreased at 95°C. It showed that gliadins were covalent-linked with glutenins and the gluten network was stronger at 95°C, which made gliadins difficult to be extracted by alcohol solution. The amount of α/β-, and γ-gliadin significantly decreased, which was more obvious than ω 5-, ω1, 2-gliadin at 95°C. It was because α/β-, and γ-gliadin were rich in free sulfhydryl groups, which could form covalent cross-linking with glutenins through disulfide bonds under heating treatment (22). However, the extra WUAX retarded the variations of the α/β-, and γ-gliadin during heating treatment compared with the G group (Table 2). This was due to the large steric hindrance of WUAX, which increased the pores in the gluten network and resulted in the solvation of gliadins. In the meantime, the reduction of α/β-, and γ-gliadin by EAX was more evident compared with the G group. This phenomenon indicated that extra EAX could promote the aggregation of glutenins and gliadins, which was realized by α/β-, and γ-gliadin at a high heating temperature. Wang et al. (32) also found that low Mw WEAX could be more effective for inducing the aggregation of glutenins and gliadins mainly by α/β-, and γ-gliadin.

**TABLE 2:** The different subunits distribution of samples at different thermal treated temperatures.

Samples		ω 5-	ω 1,2-	α -	γ -	Total gliadin	ω_*b*_-LMS	HMS	LMS	Total glutenin	Glia/glu
**G**	25°C	1.46 ± 0.06^bB^	6.24 ± 0.32^aB^	34.51 ± 0.38^cB^	25.25 ± 0.62^bB^	67.46 ± 1.39^cB^	0.52 ± 0.02^aA^	8.28 ± 0.12^cA^	23.73 ± 0.80^bA^	32.53 ± 0.94^dA^	2.07 ± 0.13^bB^
	60°C	1.48 ± 0.05^bC^	6.21 ± 0.41^bB^	33.13 ± 0.32^bC^	25.01 ± 0.39^bC^	65.83 ± 1.17^cC^	0.61 ± 0.02^cC^	9.44 ± 0.91^aB^	24.12 ± 0.60^aB^	34.17 ± 1.53^cB^	1.93 ± 0.17^cC^
	95°C	1.28 ± 0.02^bA^	5.50 ± 0.15^bA^	26.85 ± 1.06^bA^	17.71 ± 0.50^cA^	51.34 ± 1.73^cA^	0.98 ± 0.04^cC^	11.18 ± 0.69^cB^	36.49 ± 1.52^bC^	48.66 ± 2.25^cB^	1.06 ± 0.12^bA^
**GW3**	25°C	1.45 ± 0.04^bcB^	6.21 ± 0.70^aB^	35.10 ± 0.86^dB^	26.24 ± 0.30^cB^	69.00 ± 1.91^dB^	0.46 ± 0.02^aA^	7.27 ± 0.16^bA^	23.26 ± 0.52^cA^	31.00 ± 0.70^dA^	2.23 ± 0.13^cB^
	60°C	1.43 ± 0.16^bB^	5.93 ± 0.34^aB^	31.64 ± 0.72^bB^	25.1 ± 0.18^bC^	64.10 ± 1.40^cC^	0.76 ± 0.04^bC^	8.91 ± 0.73^aB^	26.23 ± 0.68^bB^	35.90 ± 1.45^dB^	1.79 ± 0.16^cC^
	95°C	1.29 ± 0.06^bA^	4.93 ± 0.35^aA^	27.43 ± 1.96^bcA^	18.8 ± 0.70^cdA^	52.46 ± 3.07^cA^	0.94 ± 0.03^cB^	9.03 ± 0.12^aA^	37.57 ± 0.94^cC^	47.54 ± 1.09^cB^	1.11 ± 0.13^bA^
**GW6**	25°C	1.46 ± 0.05^cB^	6.17 ± 0.54^cB^	35.59 ± 1.02^eC^	26.68 ± 0.52^dB^	69.90 ± 2.13^eC^	0.41 ± 0.01^aA^	6.33 ± 0.10^aA^	23.34 ± 0.38^dA^	30.09 ± 0.50^cA^	2.32 ± 0.15^dB^
	60°C	1.42 ± 0.03^bB^	6.22 ± 0.28^bB^	31.48 ± 0.96^bB^	25.15 ± 0.26^cB^	64.28 ± 1.53^dB^	0.76 ± 0.05^bB^	8.44 ± 0.45^aC^	26.52 ± 0.55^bB^	35.72 ± 1.05^bB^	1.80 ± 0.14^dC^
	95°C	1.32 ± 0.13^bA^	5.01 ± 0.37^bA^	27.27 ± 0.70^cA^	18.79 ± 1.34^dA^	52.39 ± 2.53^cA^	0.93 ± 0.03^cB^	8.83 ± 0.14^bB^	37.86 ± 0.31^dC^	47.61 ± 0.47^bB^	1.10 ± 0.09^cA^
**GE3**	25°C	1.53 ± 0.24^bB^	6.47 ± 0.52^abB^	33.36 ± 0.50^bC^	24.69 ± 0.31^bB^	66.04 ± 1.57^bC^	0.63 ± 0.04^bA^	9.42 ± 0.33^dB^	23.90 ± 0.34^bA^	33.96 ± 0.71^bA^	1.94 ± 0.14^bB^
	60°C	1.50 ± 0.06^aB^	6.27 ± 0.48^aB^	28.64 ± 0.68^aB^	23.29 ± 0.37^aB^	59.69 ± 1.60^bB^	0.89 ± 0.02^bB^	10.7 ± 0.27^aC^	28.71 ± 0.63^aC^	40.31 ± 0.92^aB^	1.48 ± 0.10^bC^
	95°C	1.19 ± 0.05^aA^	4.95 ± 0.16^aA^	24.83 ± 0.48^aA^	15.90 ± 1.09^bA^	46.87 ± 1.78^bA^	1.02 ± 0.02^bA^	12.0 ± 0.03^aA^	40.11 ± 1.09^aB^	53.13 ± 1.13^aA^	0.88 ± 0.07^aA^
**GE6**	25°C	1.47 ± 0.06^aB^	6.26 ± 0.07^aB^	33.22 ± 0.32^aB^	23.14 ± 0.37^aB^	64.09 ± 0.82^aB^	0.72 ± 0.02^bB^	10.63 ± 0.21^dA^	24.56 ± 1.04^aA^	35.91 ± 1.18^aA^	1.78 ± 0.12^aB^
	60°C	1.46 ± 0.07^aC^	6.00 ± 0.14^aC^	28.23 ± 0.53^aB^	23.22 ± 0.80^aC^	58.91 ± 1.54^aC^	1.05 ± 0.04^cC^	10.97 ± 0.64^aB^	29.06 ± 0.81^aC^	41.09 ± 1.49^aC^	1.44 ± 0.13^aC^
	95°C	1.20 ± 0.11^aA^	5.01 ± 0.34^aA^	24.00 ± 1.21^aA^	13.02 ± 0.45^aA^	43.23 ± 2.11^aA^	0.74 ± 0.03^aA^	13.98 ± 0.39^bA^	42.05 ± 1.10^aB^	56.77 ± 1.52^aB^	0.76 ± 0.08^aA^

*Data are mean ± SD (n = 3). Different lowercase letters in the same column mean significant differences in AX contents, and different capital letters in the same row mean significant differences in heating temperatures (P < 0.05). GW3, gluten with 3% (w/w) WUAX; GW6, gluten with 6% (w/w) WUAX; GE3, gluten with 3% (w/w) EAX; GE6, gluten with 6% (w/w) EAX.*

According to retention time, glutenin subunits can be divided into bound gliadin components (ωb-gliadin), high Mw glutenin subunits (HMW-GS), and low Mw glutenin subunits (LMW-GS) (33). From Table 2, all of the glutenin factions were increased with the increasing heating temperature. It was because the high temperature could induce the aggregation of glutenin subunits (34). The extra WUAX decreased the amount of HMW-GS in

gluten protein, and the effect was more significant at 95°C. Meanwhile, the extra WUAX increased the amount of LMW-GS. This might be due to the steric hindrance and physical entanglement of WUAX, which hampered the further cross-linking of glutenins molecules, thus they could only combine into LMW-GS. The extra EAX significantly increased the amount of HMW-GS and the amount of LMW-GS, in especial of LMW-GS. This phenomenon indicated that the low Mw EAX could induce the aggregation of glutenin molecules, which was mainly on LMW-GS. It has been reported that excessive aggregation of gluten molecules will lead to rigid and heterogeneous dough texture (35), so the moderate inhibition of heat-induced aggregation of gluten by EAX might be the reason why EAX led to good viscoelasticity and uniform gluten network structure.

### Aromatic Amino Acid Microenvironment

Raman spectroscopy can be used to characterize the amino acid side chain microenvironment of gluten protein molecules, which plays an important role in observing polarity and hydrogen bond changes of the protein microenvironment (23). The Raman bands near 760 cm^–1^ are due to the vibrations of the indole ring in tryptophan side chains. A decrease in band intensity at 760 cm^–1^ indicated that the tryptophan residues were exposed to a more hydrophilic environment (36). Meanwhile, the increase of band intensity was related to the increase in the burial property of Trp residues.

**FIGURE 2:**
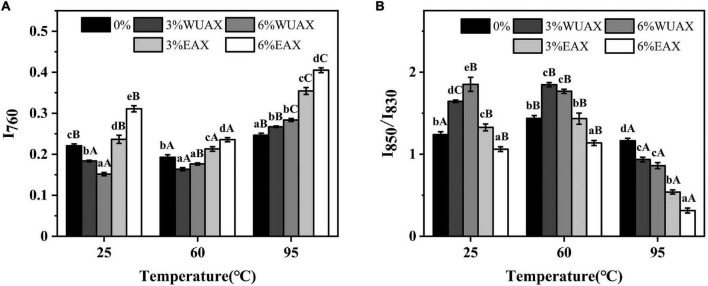
Effect of WUAX and EAX on the tertiary structure content of gluten: **(A)** I_760_ and **(B)** I_850_/I_830_. 3%WUAX, gluten with 3% (w/w) WUAX; 6%WUAX, gluten with 6% (w/w) WUAX; 3%EAX, gluten with 3% (w/w) EAX; 6%EAX, gluten with 3% (w/w) EAX.

As shown in Figure 2A, the Raman spectral intensity near 760 cm^–1^ changed obviously under heating treatment. It indicated that the hydrogen bond interaction between gluten molecules was significantly affected by heating treatment. The band intensity at 760 cm^–1^ decreased at first and then increased with increasing the temperature. This phenomenon indicated that the Trp residues were exposed firstly and then buried during heating. It was speculated that the gluten molecules unfolded and then folded and aggregated. This process involved hydrogen bonding in which Trp residues were relevant. At 25°C, extra WUAX caused the intensity of the Raman peak of 760 cm^–1^ to increase at first and then decrease, indicating that the protein unfolded by the extra WUAX and exposed the internal Trp residues. When heated at 60°C, the effect of WUAX on tryptophan residues was similar to that at 25°C. However, when heated up to 95°C, the band intensity at 760 cm^–1^ of all the samples with WUAX was higher than that of the control (without WUAX). The reason could be the unfolding of gluten, which was induced by WUAX at low-temperature heating treatment which promoted the aggregation behavior of gluten molecules at high-temperature heating treatment. Different from the WUAX, the extra EAX increased the band intensity of 760 cm^–1^ at all heating temperatures. It was indicated that extra EAX caused the gluten molecules to form a more polar microenvironment of Trp residues. It could be due to the hydroxyl groups in side chains of EAX (37). Besides, it might be because the extra EAX induced the aggregation of gluten, which caused the masking of Trp residues.

The characteristic vibration frequencies of benzene ring absorption vibration and surface bending vibration of tyrosine were near 830 and 850 cm^–1^. Tyrosine is a hydrophobic amino acid exposed to the gluten molecular surface, resulting in more hydrophobicity of gluten protein. The relative intensity (I_850_/I_830_) is always used to identify the degree of burial and exposure of Tyr residues. The decrease of I_850_/I_830_ indicated that some of the hydroxyl groups on Tyr were involved in the formation of intramolecular or intermolecular hydrogen bonds (37). As shown in Figure 2B, it could be observed that heating treatment caused a significant change in the ratio of I_850_/I_830_, indicating that the gluten conformation had changed during heating treatment. Therefore, the expansion of gluten at 60°C might have involved the exposure of tyrosine residues, while further aggregation of glutenin-glutenin and gliadin-glutenin at the higher temperature might have buried Tyr residues. With extra WUAX, the I_850_/I_830_ of gluten increased at 25°C and 60°C and decreased at 95°C compared with the control group. This changing trend of I_850_/I_830_ was opposite to that of I_760_, which further verified the conclusion of tryptophan microenvironment analysis. The extra EAX led to a decrease in I_850_/I_830_ compared with the control, indicating an increase in the buried property of tyrosine residues, that was also consistent with the analysis of tryptophan residues. According to the intensity ratio of the two bands (830 cm^–1^ and 850 cm^–1^), it could be determined whether Tyr residues were the donor or acceptor of the hydrogen bond (38). From Figure 2B, the increase of I_850_/I_830_ with extra WUAX indicated that the Tyr residues were trend to act as proton acceptors in the interaction between gluten and WUAX. However, the decrease of I_850_/I_830_ with extra EAX indicated that the Tyr residues tended to act as proton donors in the interaction between gluten and EAX.

### Characterization of Gluten Microstructure by CLSM

**FIGURE 3:**
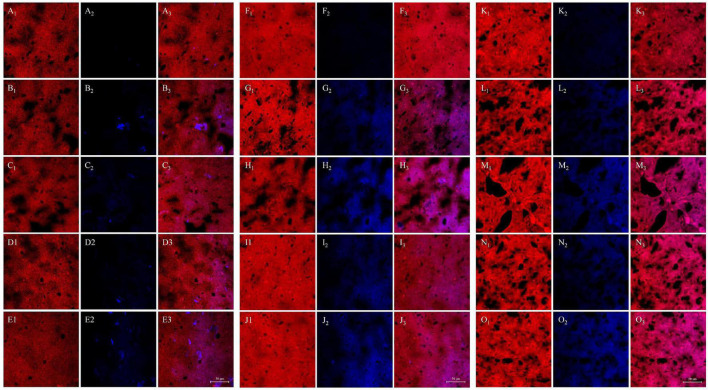
Influence of WUAX and its hydrolysates on gluten microstructure at different thermal treated temperatures by CLSM. **(A–E)** The temperature of 25°C. **(F–J)** The temperature of 60°C. **(K–O)** The temperature of 95°C. The subscript number 1 was the visualized proteins (the red color). The subscript number 2 was the visualized AXs (the blue color). The subscript number 3 was the merged image combined with protein and AXs. Each row represented G, GW3, GW6, GE3, and GE6, respectively. G, gluten; GW3, gluten with 3% (w/w) WUAX; GW6, gluten with 6% (w/w) WUAX;GE3, gluten with 3% (w/w) EAX; GE6, gluten with 6% (w/w) EAX.

The influence of WUAX and its hydrolysates on gluten microstructure at different thermal treatment temperatures was identified by CLSM, the results are presented in Figure 3 (red color for gluten protein and blue color for AX). It could be seen that the untreated gluten sample showed a uniform network structure. However, the GW group appeared as large and irregular voids in the gluten network at 25°C. It was indicated that the presence of WUAX destroyed the original uniform network structure of gluten protein and formed a dispersed network structure. However, in contrast with the G and GW groups, there was a compact gluten network in the GE group, which stabilized the gluten network and form a more uniform and dense network structure. This showed that EAX could improve the gluten network structure, reduce the voids, and the gluten molecules had more extensive cross-linking. The CLSM images showed that the large and irregular voids still appeared in the gluten network with extra WUAX at 60°C. When the heating temperature went up to 95°C, the network structure of gluten with extra WUAX was destroyed more seriously, the voids were more and larger, the protein framework was broken and the continuous structure was damaged and incomplete. Relatively speaking, the gluten network structure with extra EAX had more voids than the G group at 95°C, but these voids were small and regular. Furthermore, the network structure was homogenous and dense, and the cross-linking between gluten chains was more extensive. It indicated that the extra EAX could stabilize the network structure of gluten and support the cross-linking of protein molecular chains even under high heating temperature denaturation conditions. It could be speculated that WUAX had a higher molecular weight and long molecular chain, thus it reduced the contact of gluten and hindered the cross-linking between gluten molecules. While the molecule chain of the EAX was disrupted by xylanase, the EAX with low Mw might have interspersed in the gluten network and bridge protein molecules. These phenomena were similar to that of Li et al. (39), who demonstrated that AX with higher Mw caused the instability of dough, and AX factions with medium Mw fully interacted with gluten to form a uniform network structure. Moreover, compared with the samples at 25°C, the samples after heating treatment had no obvious blue spots, but showed a wide range of blue areas, indicating that AX factions infiltrated into the gluten network and molecular structure and co-located with gluten protein after heating treatment.

### Interactions Between Arabinoxylans and Gluten

The interactions between AX and gluten include hydrophobic interaction (Δ H > 0 and Δ S > 0), van der Waals force, hydrogen bond (if Δ H < 0 and Δ S < 0), and electrostatic interaction (Δ H < 0 and Δ S > 0) (25). A positive Δ H indicated that the interactions between AX and gluten were an endothermic binding process, while a positive Δ S value indicated an increase in disorder of the system (24). As shown in Table 3, in three temperature ranges, the Δ G of gluten protein is negative, indicating that the reaction process between WUAX with gluten as well as EAX with gluten was spontaneous (40). The system was negative Δ H and negative Δ S at three different heating temperatures, so it could be considered that there was mainly van der Waals force and hydrogen bond between WUAX and gluten molecules. This phenomenon showed that the interactions between WUAX and gluten remained stable during heating treatment. However, the system was positive Δ H and positive Δ S at 25°C and 60°C, but it was negative Δ H and negative Δ S at 95°C. It showed that the interactions between EAX and gluten were mainly hydrophobic interaction at 25°C and 60°C, and they were mainly van der Waals force and hydrogen bond. The results showed that WUAX and EAX interacted with gluten protein in different ways during heat treatment, which had different effects on gluten protein.

**TABLE 3:** Thermodynamic parameters obtained for gluten-AX interactions from fluorescence spectroscopy.

Temperature (K)	WUAX			EAX		
	Δ G (KJ⋅mol^–1^)	Δ S (KJ⋅mol^–1^⋅K^–1^)	Δ H (KJ⋅mol^–1^)	Δ G (KJ⋅mol^–1^)	Δ S (KJ⋅mol^–1^⋅K^–1^)	Δ H (KJ⋅mol^–1^)
293	−30.30	−0.25	−103.6	−28.50	0.62	153.2
298	−29.04			−31.60		
303	−27.79			−34.70		
328	−48.12	−1.61	−576.3	−28.30	0.91	270.1
333	−40.06			−32.85		
338	−32.01			−37.40		
363	−43.86	−0.24	−129.6	−33.97	−0.49	−210.1
368	−42.68			−31.55		
373	−41.50			−29.12		

*WUAX, water-unextractable arabinoxylan; EAX, enzymatic hydrolysis arabinoxylan.*

## Conclusion

This present study evaluated the influence of water-unextractable arabinoxylan and its hydrolysates on the aggregation and structure of gluten proteins. The results showed that the enzymatic degradation significantly reduced the Mw of AX. Through different subunits distribution assays, WUAX hindered the formation of high Mw glutenin subunits because of its macromolecular chain, while the EAX induced the aggregation of gluten molecules, especially the low Mw glutenin subunits. Combined with FT-Raman analysis, the WUAX and EAX were involved in the unfolding and folding process during heating treatment, and EAX could form hydrogen bonds with the tyrosine residues in gluten molecules. The CLSM results showed that the presence of WUAX destroyed the original uniform network structure of gluten protein and formed a dispersed network structure. But the extra EAX could stabilize the network structure of gluten and support the cross-linking of protein molecular chains even under high heating temperature denaturation conditions. Our finding indicated that the enzymatic hydrolysis can weaken the negative effect of WUAX on the gluten network. Meantime, the small molecule AX produced by enzymatic hydrolysis helps the formation of gluten network structure, thus improving the quality of whole wheat products.

## Data Availability Statement

The original contributions presented in the study are included in the article/supplementary material, further inquiries can be directed to the corresponding author.

## Author Contributions

JS: conceptualization, methodology, data curation, software, and writing – original draft preparation. XS, TL, and JZ: conceptualization and data curation. HQ: reviewing and editing. YL: data curation and project administration. HZ and XQ: validation. LW: conceptualization, reviewing and editing, funding acquisition, and writing – original draft preparation. All authors contributed to the article and approved the submitted version.

## Conflict of Interest

The authors declare that the research was conducted in the absence of any commercial or financial relationships that could be construed as a potential conflict of interest.

## Publisher’s Note

All claims expressed in this article are solely those of the authors and do not necessarily represent those of their affiliated organizations, or those of the publisher, the editors and the reviewers. Any product that may be evaluated in this article, or claim that may be made by its manufacturer, is not guaranteed or endorsed by the publisher.
